# Medich Giant Platelet Syndrome: An Evolving Qualitative and Quantitative Platelet Disorder

**DOI:** 10.3390/hematolrep14040049

**Published:** 2022-12-01

**Authors:** Gita Massey, Laura Tyrrell, Yaser Diab, William T. Gunning

**Affiliations:** 1 Division of Pediatric Hematology and Oncology, Department of Pediatrics, Virginia Commonwealth University School of Medicine, Richmond, VA 23298, USA; 2 Indiana Hemophilia and Thrombosis Center, Indianapolis, IN 46260, USA; 3 Division of Pediatric Hematology, Children’s National Medical Center, Washington, DC 20010, USA; 4 Department of Pathology, University of Toledo College of Medicine and Life Sciences, Toledo, OH 43614, USA

**Keywords:** thrombocytopenia, platelet dysfunction, macro-thrombocytopenia, alpha granules

## Abstract

Qualitative platelet disorders remain rare and varied. We describe here 2 additional patients with giant platelets, thrombocytopenia, deficiency in alpha granules and the presence of membranous inclusions within the cytoplasm. Collectively known as Medich syndrome, we further elucidated structural and clinical features of this rare syndrome. Platelets obtained from 2 patients with macro-thrombocytopenia were evaluated by electron microscopy. Structural findings were correlated with clinical characteristics. The defining morphologic feature found in the platelets of these patients is the presence of long, tubular inclusions consisting of several layers of membrane wrapped around a core of cytoplasm. These inclusions may deform the discoid shape of the platelet. In addition, abnormal giant alpha granules are present. Clinically all patients in the current report and review of the literature had mucosal bleeding and were often misdiagnosed as having immune related thrombocytopenia. To date five cases of Medich giant platelet syndrome have been reported. The cases are unified by the ultrastructural findings of abnormal alpha granules and unusual cytoplasmic scrolls. All patients experienced mucosal bleeding, however many clinical, biologic and genetic characteristics of this rare disorder remain to be determined.

## 1. Introduction

Qualitative platelet disorders encompass a wide range of clinical syndromes. Specifically, human platelet granule deficiency disorders are relatively rare, but quite varied and often associated with other physical stigmata [1,2]. These disorders include gray platelet syndrome with decreased numbers to absence of alpha granules [3,4] and Hermansky-Pudlak syndrome [5], Chediak-Higashi syndrome [6], and Wiskott Aldrich syndrome [7] which are the best known conditions with absence or decreased numbers of dense granules. Additional dense granule deficiency disorders include Jacobsen/Paris Trousseau syndrome [8], thrombocytopenia with absent radius syndrome (TAR syndrome) [9], familial platelet disorder with RUNX1 mutations [10] that, and alpha-delta granule storage pool deficiency and white platelet syndrome that have decreased numbers of both alpha and dense granules) [11,12,13].

In 2004, a patient with giant platelets and thrombocytopenia was reported to have decreased numbers of alpha granules and the presence of membranous inclusions resembling “cigars” or “scrolls” when evaluated by electron microscopy [14]. These inclusions had not previously been reported in any other human platelet disorder, but are common in the Wistar-Furth rat platelets [14]. In 2013, two additional unrelated patients were described with platelet pathology identical to that of the index case [15]. We now report two additional patients with similar platelet ultrastructural features, thus further adding to the descriptive features of a new syndrome; the Medich giant platelet syndrome.

## 2. Materials and Methods

### 2.1. Patients

*Case 1:* A Hispanic female born full-term with no known prenatal or perinatal complications presented at one month of age with aseptic meningitis. She had thrombocytopenia with platelet count of 12,000/mL. She was thought to have idiopathic thrombocytopenic purpura (ITP). Peripheral smear revealed large platelets. Comprehensive diagnostic studies over several years included a normal bone marrow biopsy and multiple light transmission aggregometry (LTA) studies using the agonists adenosine diphosphate (ADP), epinephrine, collagen, arachidonic acid, and ristocetin were deemed inconclusive because of her persistent thrombocytopenia. Her PFA Collagen/epi and PFA Collagen/ADP closure times were both normal at 106 s and 88 s, respectively with a platelet count of 58 K. Platelet Glycoprotein expression was sent to then Blood Center of Wisconsin for assay of GPIb and GPIIb/IIIa which was also reported as normal. We did not pursue additional flow studies once the platelet EMs identified Medich inclusions. Additional entities that were ruled out included vascular malformations, type 2B von Willebrand disease, congenital thrombotic thrombocytopenic purpura, and autoimmune lymphoproliferative syndrome. She received multiple therapeutic interventions including, steroids, immunoglobulin infusions, mycophenolate, anti-CD20 immunotherapy, mTOR inhibitor sirolimus, and splenectomy, but consistently responded best to platelet transfusions. She also had a microcytic anemia that did not correct completely with iron therapy. Alpha chain DNA analysis revealed duplication of an alpha globulin gene on one allele. At the age of 8 years, platelet morphology was evaluated by electron microscopy and inclusions in her platelets were consistent with Medich giant platelet syndrome. Since her diagnosis, her platelet count has ranged between 30,000/mL to 60,000/mL but has dropped on rare occasion to <10,000/mL with associated illness. Bleeding symptoms have been mostly bruising and some episodes of mucosal bleeding (epistaxis, significant hematoma with a dog bite requiring sutures). At puberty, menorrhagia was a significant problem which has been controlled with depoprovera. For acute bleeding episodes, platelet infusions have always helped. Antifibrinolytics such as aminocaproic acid and more recently tranaxemic acid have also been used.

*Case 2*: An African American male born full-term with no known prenatal or perinatal complications was found to have moderate thrombocytopenia (platelet count of 60,000/mL) at birth. His mother was thrombocytopenic during pregnancy. Review of his peripheral smear showed mostly normal sized granulated platelets and Dohle-body like inclusions were seen in neutrophils. Testing for neonatal allo-immune thrombocytopenia was negative and he was initially diagnosed with neonatal ITP due to presumed maternal ITP. He did not have significant bleeding issues during infancy and early childhood but was followed closely and his thrombocytopenia persisted (platelet count range 50,000–61,000/mL) raising concerns for possible familial thrombocytopenia. Aggregation assays and flow cytometry were not performed due his lack of significant bleeding history and his persistent thrombocytopenia. At the age of 5 years, he experienced severe oral mucosal bleeding that resolved after receiving platelet transfusion and aminocaproic acid. At the age of 6 years, platelet morphology was evaluated by electron microscopy that revealed findings consistent with Medich giant platelet syndrome.

### 2.2. Platelet Preparation Methods for Electron Microscopy

Preparation of whole-mounted platelets was performed by established techniques in the published literature [16,17,18]. Briefly, blood was obtained by venipuncture and drawn into acid citrate dextrose BD Vacutainer^®^ blood collection tubes (Sol B, Becton, Dickinson, Franklin Lakes, NJ, USA). Platelet-rich plasma (PRP) was obtained by centrifugation at room temperature for 15 min at 100× *g*. Twenty microliter drops of PRP were placed upon parlodion coated 300 mesh copper grids and incubated for 5 min. Grids were washed with deionized water for 10 s. and air-dried. Whole mounted platelets were viewed with a FEI Tecnai T20 transmission electron microscope using 80 KV for the accelerating voltage. Platelet dense granules were enumerated in a total of 100 consecutive whole-mounted platelets, except for those partially obscured by a grid support bar, to obtain an average number of dense granules per platelet. Previous studies from our laboratory have established a normal range of 4.60 + 0.47 DG/PL [18] which is similar to ranges also published in the literature [5,19,20]. Once aliquots of PRP were acquired for the preparation of platelet whole mounts, an additional centrifugation was performed to compact the buffy coat; the platelet poor plasma was decanted and 3% glutaraldehyde buffered with 0.2 M sodium cacodylate buffer (pH 7.2) was layered upon the buffy coat in the vacutainer tube for primary overnight fixation. Subsequently, the fixed buffy coat was gently removed from the vial, minced with a razor blade as if a solid tissue, and washed with 0.2 M cacodylate buffer. Secondary fixation utilized reduced osmium (1% osmium tetroxide and 1.5% potassium ferricyanide in dist. H_2_O) for 1 h [21], washed with H_2_O_2_, followed by a tertiary fixation step using saturated aqueous uranyl acetate for 1 h, dehydrated, embedded, and thin sectioned for transmission electron microscopy (TEM) to evaluate platelet morphology.

**Figure 1:**
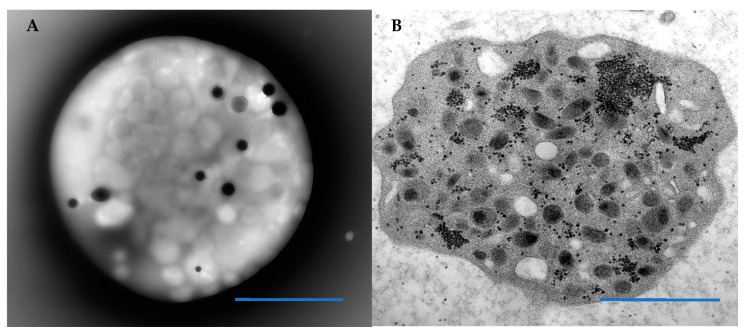
The ultrastructural morphology of platelets can be assessed by either a whole mounted, air-dried technique that allows enumeration of platelet dense granules. (**A**) A normal platelet with 10 dense granules. Platelets may also be sectioned in epoxy embedded buffy coat to evaluate organelle morphology and alpha granule content such as the platelet depicted in (**B**) that has numerous alpha granules and aggregates of black granular glycogen. Blue Bars = 1 micron.

Preparation of thin sections from the buffy coat for evaluation of platelet morphology allows for observation of literally thousands of platelets and hundreds of nucleated white cells. With the exception of gray platelet syndrome that may be essentially devoid of alpha granules, most platelet syndromes have a preponderance of platelets with normal morphology even if platelet size may vary. Therefore, we utilize platelets with normal morphology in our sections of buffy coat as internal standards (Figure 1). The use of normal donor blood for comparison of platelet morphology is not required, nor was used in the diagnosis of Medich syndrome. The scrolls first reported White that we also describe have never been reported in any other publication of macrothrombocytopenia.

Normal circulating platelets resemble flattened discs. The discoid shape is maintained by a circumferential coil of microtubules lying just below the surface membrane (Figure 1). The platelet cytoplasm is filled with organelles, of which the most common are alpha granules. Lysosomes, mitochondria, and dense bodies (also known as delta granules, the storage sites for adenine nucleotides, calcium, pyrophosphates, and serotonin) are also present in variable numbers, as is glycogen. Two channel systems are present. The open canalicular system consists of convoluted and interconnected conduits derived from the surface membrane. The channels of the dense tubular system originate from the rough and subsequently smooth endoplasmic reticulum of the megakaryocyte.

## 3. Results

### 3.1. Patient Platelets

**Figure 2:**
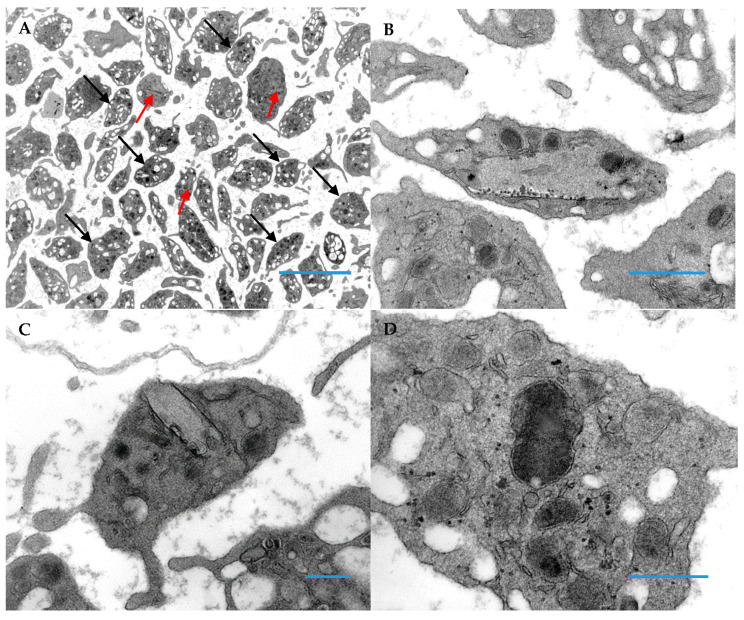
Platelets sectioned from epoxy embedded buffy coat of patient 1. (**A**) Unusual inclusions (red arrows) are almost impossible to visualize at low magnifications; black arrows indicate normal platelets (Original magnification 3000×; Blue Bar = 10 microns). (**B**) An inclusion occupies most of the platelet cytoplasm (Original magnification 13,000×; Blue Bar = 1 micron). (**C**) An inclusion appears to merge with the cell membrane; this represents an inclusion that deforms the platelet’s shape and “sticks-out” from the cell (this section is just above/below of the protrusion “out” of the platelet (Original magnification 13,000×; Blue Bar = 1 micron). This type of inclusion is easily identified in platelet whole mounts. (**D**) This image demonstrates a giant alpha granule created by fusion of 2 or more alpha granules and is characteristic of Medich syndrome (Original magnification 30,000×; Blue Bar = 1 micron); these giant alpha granules are similar to those seen in chromosome 11q deletion syndrome (Jacobsen/Paris-Trousseau syndromes).

**Figure 3:**
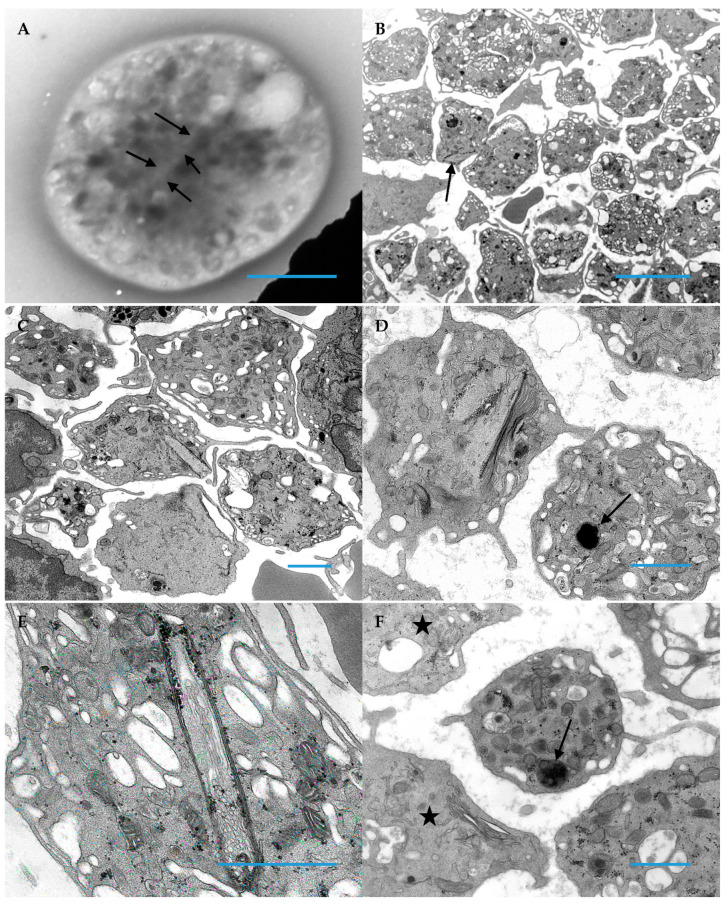
Platelets from patient 2 demonstrate the character inclusions common for Medich syndrome. (**A**) This image demonstrates a faint outline (arrows) in a whole mounted, air-dried platelet; these are almost impossible to visualize when entirely within a cell, unlike inclusions that protrude from the cell and create a stark deformation of the discoid platelet shape (Original magnification 11,000×; Blue Bar = 1 micron). (**B**) Macrothrombocytes having vacuolated cytoplasm are readily apparent with one having a peripheral inclusion (arrow) (Original magnification 4800×; Blue Bar = 10 microns). (**C**) An inclusion in the center of this image is protruding from a platelet (Original magnification 8000×; Blue Bar = 1 micron). (**D**) Adjacent platelets demonstrate the two morphologic characteristics of Medich syndrome; one platelet has a giant alpha granule (arrow), whereas the second platelet has 2 scroll shaped inclusions (Original magnification 13,000×; Blue Bar = 1 micron). (**E**) The image demonstrates black granular aggregates of glycogen associated the multi-membranous “scroll” inclusion in a grey platelet (Original magnification 30,000×; Blue Bar = 1 micron). (**F**) In this image, two gray platelets (stars) absent of alpha granules are demonstrated and an additional platelet has a giant alpha granule (arrow) in contrast to the normal size of alpha granules populating the platelet (Original magnification 11,000×; Bar = 1 micron).

Thin sections of platelets from the two patients are shown below (Figure 2 and Figure 3). The majority of platelets of both subjects had an appearance of vacuolation, likely due to expansion of the open canalicular system. Some of the platelets were large and devoid of formed organelles, especially in case 2 in which alpha granules were not present and similar to the morphology of gray platelets (Figure 3C,D). They also contain scroll-like structures in early stages of formation. In more mature thrombocytes these scroll-like structures are long and stretch across the cytoplasm resulting in deformation of the thrombocyte. These structures appear to be associated with aggregates of glycogen. An additional observation was abnormally fused alpha granules in occasional platelets, forming giant alpha granules as demonstrated in Figure 2D and Figure 3D,F.

### 3.2. Clinical Characteristics of Patients

Table 1 is a summary of the essential clinical characteristics of the original index case of Medich reported by White [14], the subsequent report of two additional cases by Gunning et al. [15], and the two cases presented in this report. The common features of the previously reported Medich syndrome cases as well as our two cases include the presence of thrombocytopenia in the first year of life, misdiagnosis as immune related thrombocytopenia, and the presence of mucosal bleeding that responds to platelet transfusions. One of our cases also had whole exome sequencing done (Ambry Genetics) that identified a de novo missense variant in the SLFN14 gene, c.652A > G.

## 4. Discussion

The current manuscript reports two additional patients with Medich syndrome whose platelets exhibit the same features as the three previously described Medich patients [14,15]. The common presentation for all five cases included thrombocytopenia at or near birth that persisted without resolve. For most, the diagnosis took years and required the use of electron microscopy to identify abnormal, giant alpha granules as seen in chromosome 11 q deletion syndrome, formally known as Jacobsen/Paris-Trousseau syndromes [8] as well as long scroll-like structures that stretch across platelet cytoplasm, often resulting in deformation of the thrombocyte [14,15]. Many of the giant platelet disorders may be diagnosed with clinical history and subsequent macrothrombocytopenia such as immune thrombocytopenic purpura (ITP) following respiratory infection. Other conditions such as Glazmann thrombasthenia and Bernard-Soulier syndromes can be diagnosed by flow cytometry or molecular techniques as these disorders have well established molecular mutations [22,23,24,25]. A recent review by Collins et al. reports that there are two phenotypically distinct macrothrombocytopenic disorders, white platelet and Medich platelet syndromes for which a genetic alteration has yet to be identified [26]. Unfortunately, we are unable to provide identification of a specific gene other than the duplication of the alpha globin gene in one allele. This anomaly has been seen a number of times in patients without macrothrombocytopenia or other platelet disorders by one of the authors and therefore considered an incidental observation.

With the exception of Case 2 (who had normal sized platelets on peripheral smear), all patients had a deficiency of alpha granules in giant platelets (>5 µm) but not in normal sized platelets (1–2 µm). The giant platelets also had more cytoplasmic vacuoles than alpha granules, mitochondria, and dense granules as seen in Figure 3B. This may be due to an expansion of the open canalicular system. Smaller, normal sized platelets had a normal component of organelles. In addition, the subjects of this report had abnormal giant alpha granules found in both normal sized and giant platelets, similar to the characteristic morphologic change seen in Jacobsen’s/Paris-Trousseau syndrome and the previously described Medich syndrome patients [8,14,15].

However, the defining morphologic feature found in the platelets of these patients is the presence of long, tubular inclusions consisting of several layers of membrane wrapped around a core of cytoplasm open at both ends. In some instances, these inclusions deform the discoid shape of the platelets. Previous freeze-fracture studies have shown that these membranous scrolls are devoid of intramembranous particles, a finding not previously seen in any human normal or abnormal platelets [14]. These scrolls in early stages of development were empty, but as they developed, glycogen particles filled their cores of cytoplasm. In the longer scrolls, these particles were attached to the innermost membrane surface. The origin of these inclusions remains unknown as does their impact on platelet function and number.

Indeed, many clinical, biologic, and importantly, genetic characteristics of Medich syndrome remain to be determined. Patient demographics for our current patients (cases 1 and 2) and the previously described cases of Medich syndrome (case 3 is the original index patient [14] and cases 4 and 5 were reported in 2013 [15]) are summarized in Table 1. The common clinical findings include symptoms suggestive of bleeding due to qualitative or quantitative platelet disorders and subsequently the diagnosis of macro-thrombocytopenia during the first year of life (most commonly in the neonatal period). These bleeding symptoms are similar to other platelet quantitative and qualitative disorders and include bruising, epistaxis and other mucosal bleeding, such as heavy menstrual bleeding. A life threatening bleed (subdural hematoma) was reported in one patient. Response to platelet transfusions suggests that this is not an immune mediated process.

Patient 1 did have whole exome sequencing (Ambry Genetics) which identified a de novo missense variant in the SLFN14 gene, c.652A > G. This variant in exon 1 of the ATPase-AAA domain has not been reported in large exome databases, but is likely pathogenic as SLFN14 (located at 17q12 is thought to be involved in megakaryocyte maturation and platelet formation [27,28].

Interestingly, two of five patients also had additional abnormalities which included a 46 XY deletion (11q23.3) and a duplication of the alpha globin gene in one allele. Current technologies such as whole genome sequencing may help define the etiology of Medich syndrome, while a formal registry of such patients could elucidate the natural history and long-term implications of Medich syndrome.

## Figures and Tables

**Table 1:** Clinical Findings in Patients with Reported Medich Syndrome. + OCP; oral contraceptives; ++ CHD: congenital heart disease; +++ EGA; estimated gestational age.

Patient	Sex	Ethnicity	Age of Initial Thrombocytopenia	Age at Diagnosis	Bleeding Symptoms	Therapies	Other Findings	Ref.
1	Female	Hispanic	2 weeks	8 years	Bruising Mucosal bleeding	IVIG Steroids Anti-CD20 Immunosuppressants Platelet transfusion Splenectomy Antifibrinolytics OCP+	Duplication of alpha globin gene in one allele Missense in the SLFN14 gene, c.652A > G	
2	Male	African American	Birth	6 years	Bruising Oral mucosal bleeding	Platelet transfusion Antifibrinolytics	Maternal thrombocytopenia	
3	Female	Caucasian	1 year	19 years	Bruising Bleeding with piercings and dental work Menorrhagia	Platelet transfusion Splenectomy OCP+ Antifibrinolytics	None	[14]
4	Male	Caucasian	Birth	2 years		Platelet transfusion	CHD++ Craniosynostosis 4Ydel (11q23.3)	[15]
5	Female	Caucasian	Birth	18 months	Subdural hematoma	Platelet transfusion	EGA+++ 29 weeks one of fraternal triplets	[15]

## Data Availability

All data that is available is contained within the body of this manuscript.

## References

[1] Cox K., Price V., Kahr W.H. (2011). Inherited platelet disorders: A clinical approach to diagnosis and management. Expert Rev. Hematol..

[2] Israels S.J., Kahr W.H., Blanchette V.S., Luban N.L., Rivard G.E., Rand M.L. (2011). Platelet disorders in children: A diagnostic approach. Pediatr. Blood Cancer.

[3] Nurden A.T., Nurden P., Bermejo E., Combrie R., McVicar D.W., Washington A.V. (2008). Phenotypic heterogeneity in the Gray platelet syndrome extends to the expression of TREM family member, TLT-1. Thromb. Haemost..

[4] White J.G. (1979). Ultrastructural studies of the gray platelet syndrome. Am. J. Pathol..

[5] Witkop C.J., Krumwiede M., Sedano H., White J.G. (1987). Reliability of absent platelet dense bodies as a diagnostic criterion for Hermansky-Pudlak syndrome. Am. J. Hematol..

[6] Buchanan G.R., Handin R.I. (1976). Platelet function in the Chediak-Higashi syndrome. Blood.

[7] Clauser S., Cramer-Borde E. (2009). Role of platelet electron microscopy in the diagnosis of platelet disorders. Semin. Thromb. Hemost..

[8] White J.G. (2007). Platelet storage pool deficiency in Jacobsen syndrome. Platelets.

[9] Zahavi J., Gale R., Kakkar V.V. (1981). Storage pool disease of platelets in an infant with thrombocytopenic absent radii (TAR) syndrome simulating Fanconi’s anaemia. Haemostasis.

[10] Glembotsky A.C., Bluteau D., Espasandin Y.R., Goette N.P., Marta R.F., Marin Oyarzun C.P., Korin L., Lev P.R., Laguens R.P., Molinas F.C. (2014). Mechanisms underlying platelet function defect in a pedigree with familial platelet disorder with a predisposition to acute myelogenous leukemia: Potential role for candidate RUNX1 targets. J. Thromb. Haemost..

[11] Hayward C.P., Weiss H.J., Lages B., Finlay M., Hegstad A.C., Zheng S., Cowie A., Massé J.M., Harrison P., Cramer E.M. (2001). The storage defects in grey platelet syndrome and alphadelta-storage pool deficiency affect alpha granule factor V and multimerin storage without altering their proteolytic processing. Br. J. Haematol..

[12] White J.G., Keel S., Reyes M., Burris S.M. (2007). Alpha-delta platelet storage pool deficiency in three generations. Platelets.

[13] White J.G. (2005). Golgi complexes in hypogranular platelet syndromes. Platelets.

[14] White J.G. (2004). Medich giant platelet disorder: A unique alpha granule deficiency I. Structural abnormalities. Platelets.

[15] Gunning W., Dole M., Brecher M., White J.G. (2013). The Medich giant platelet syndrome: Two new cases. Platelets.

[16] Bull B.J. (1966). The ultrastructure of negatively stained platelets. Blood.

[17] White J.G., Edson J.R., Desnick S.J., Witkop C.J. (1971). Studies of platelets in a variant of the Hermansky-Pudlak syndrome. Am. J. Pathol..

[18] Gunning W.T., Raghavan M., Calomeni E.P., Turner J.N., Roysam B., Roysam S., Smith M.R., Kouides P.A., Lachant N.A. (2020). A Morphometric Analysis of Platelet Dense Granules of Patients with Unexplained Bleeding: A New Entity of Microgranular Storage Pool Deficiency. J. Clin. Med..

[19] White J.G. (2002). Electron dense chains and clusters in human platelets. Platelets.

[20] Weiss H.J., Lages B., Vicic W., Tsung L.Y., White J.G. (1993). Heterogeneous abnormalities of platelet dense granule ultrastructure in patients with congenital storage pool deficiency. Br. J. Haematol..

[21] Lewinson D. (1989). Application of the ferrocyanide-reduced osmium method for mineralizing cartilage: Further evidence for the enhancement of intracellular glycogen and visualization of matrix components. Histochem. J..

[22] Fletcher S.A., Johnson B., Lowe G.C., Bem D., Drake S., Lordkipanidzé M., Guiú I.S., Dawood B., Rivera J., Simpson M.A. (2015). SLFN14 mutations underlie thrombocytopenia with excessive bleeding and platelet secretion defects. J. Clin. Investig..

[23] Lambert M.P. (2019). Inherited Platelet Disorders: A Modern Approach to Evaluation and Treatment. Hematol. Oncol. Clin. North Am..

[24] Pluthero F.G., Kahr W.H.A. (2019). Recent advances in inherited platelet disorders. Curr. Opin. Hematol..

[25] Kim B. (2022). Diagnostic workup of inherited platelet disorders. Blood Res..

[26] Collins J., Astle W.J., Megy K., Mumford A.D., Vuckovic D. (2021). Advances in understanding the pathogenesis of hereditary macrothrombocytopenia. Br. J. Haematol..

[27] Marconi C., Di Buduo C.A., Barozzi S. (2016). SLFN14-related thrombocytopenia: Identification within a large series of patients with inherited thrombocytopenia. Thromb. Haemost..

[28] Savoia A. (2016). Molecular basis of inherited thrombocytopenias: An update. Curr. Opin. Hematol..

